# Weather Effects on Mobile Social Interactions: A Case Study of Mobile Phone Users in Lisbon, Portugal

**DOI:** 10.1371/journal.pone.0045745

**Published:** 2012-10-10

**Authors:** Santi Phithakkitnukoon, Tuck W. Leong, Zbigniew Smoreda, Patrick Olivier

**Affiliations:** 1 Culture Lab, School of Computing Science, Newcastle University, Newcastle, United Kingdom; 2 Sociology and Economics of Networks and Services Department, Orange Labs, France; Hungarian Academy of Sciences, Hungary

## Abstract

The effect of weather on social interactions has been explored through the analysis of a large mobile phone use dataset. Time spent on phone calls, numbers of connected social ties, and tie strength were used as proxies for social interactions; while weather conditions were characterized in terms of temperature, relative humidity, air pressure, and wind speed. Our results are based on the analysis of a full calendar year of data for 22,696 mobile phone users (53.2 million call logs) in Lisbon, Portugal. The results suggest that different weather parameters have correlations to the level and character of social interactions. We found that although weather did not show much influence upon people's average call duration, the likelihood of longer calls was found to increase during periods of colder weather. During periods of weather that were generally considered to be uncomfortable (i.e., very cold/warm, very low/high air pressure, and windy), people were found to be more likely to communicate with fewer social ties. Despite this tendency, we found that people are more likely to maintain their connections with those they have strong ties with much more than those of weak ties. This study sheds new light on the influence of weather conditions on social relationships and how mobile phone data can be used to investigate the influence of environmental factors on social dynamics.

## Introduction

Weather has been the subject of studies in many fields because of its relation to, and influence on, a number of phenomena related to human behavior. Many socioeconomic activities are strongly related to weather and climate [1]. People may choose to move and live in places because of particular climate and weather sequences in order to gain personal satisfaction and for a sense of well-being. Particular places may also be attractive to people because the climate and weather is conducive to economic activities. Tucker and Gillian [2] concluded from their comprehensive literature review that physical activity vary with seasonality and identified poor or extreme weather condition as a barrier to physical activity. The levels of physical activity vary by geographical region. For example in Scotland, The Netherlands, France, Canada, the levels of physical activity appear to be highest in summer and spring (April–August). However, the levels of physical activity are lowest in July in Texas (USA) as it is the hottest month. People's thermal comfort thus plays an important role in their participation of physical activity. Stathopoulos et al. [3] found that air temperature is the dominant factor in influencing human comfort, but that wind speed, relative humidity, and solar radiation may also contribute to human perception of overall comfort. There are also significant gender differences in the perception of thermal comfort. Females have been found to be more sensitive to temperature levels, and less sensitive to humidity levels, than males [4]–[5].

Weather has also been shown to impact people's mood. Humidity, temperature and hours of sunshine had the greatest effect on mood. High levels of humidity lowered concentration, increased sleepiness while rising temperatures lowered anxiety and skepticism [6]. The relationship between attempted or completed suicide and weather or climate is also observed. While researchers have yet to identify a specific weather condition associated with a generally higher risk for suicide, interactions between weather and seasonal effects, as well as environmental effects on brain functions and weather-related interactions show a contributory role [7]. Epidemiological data also found correlations between small positive air ionization due to changing weather conditions with increases in industrial and automobile accidents, suicide, crime, depression, irritability, and interference with central nervous functions [8]. Air ionization arising from electrical and electromagnetic changes in the atmosphere are in part caused by the weather. Together with other factors such as temperature, humidity, noise, and so on, changes in ionization can affect our physical and psychological health [9]. Changes in weather have also been linked to public health. Patz et al. [10] discuss climatic variability as a determinant of disease and its importance in the construction of predictive models to support public health prevention strategies and programs.

Through its impact on human behavior, weather has also been identified as a determinant of stock prices. Saunder Jr. [11] suggested including behavioral variables influenced by weather conditions in models of asset pricing. Akhtari [12] also found a strong relationship between weather (hours of sunshine) and stock prices. He offered that particular investors' misattribution of good mood on sunny days led to biased decision-making processes that in turn influenced the overall stock prices.

Cohn's extensive literature review [13] noted a correlation between assaults, burglary, collective violence, domestic violence, and rape to changes in temperature. Such violent behaviors and crime tend to increase with ambient temperature, at least up to about 85°F. However, the relationship between heat and homicide is uncertain. In general, it appears that most violent crimes against persons increase linearly with heat, while property crimes are not strongly related to temperature changes. The mediating factors proposed to explain the relationship between heat and violent criminal behavior includes alcohol consumption, vacations, leisure time, and the availability of social interaction. This is because alcohol consumption tends to increase when the temperature is high, while vacations also generally occur during periods of warm weather increasing the potential for social interactions, especially with family and friends. Furthermore, uncomfortable heat also tends to cause increased frustration, reducing one's tolerance for annoyances.

Weather also plays an important role in determining travel behavior and traffic demands. Cools et al. [14] show that travel patterns are strongly related to weather conditions, while changes in travel behavior caused by the weather are also dependent upon the purpose of the trip. Maze et al., [15] describe the influence of inclement weather on traffic safety, traffic demand, and traffic flow, while Agarwal et al., [12] discuss how changes in weather type (rain, snow, visibility) and intensities impact the speed, headway, and capacity of roadways.

While the above review clearly affirms the importance of understanding the relationship of weather on human behavior, it is also clear that we need to find other ways that can help further shed light into this complex phenomenon. Inquiring into how weather shapes social interaction is crucial to understanding the weather's relationship to human behavior. However, we have yet to find any significant studies that have looked at this particular relationship.

To offer a way to investigate this twin concept of weather and human behavior and to establish an emergent understanding of this relationship, we describe an approach that leverages the recent widespread adoption of mobile phones, in particular its use as a platform for conducting large scale longitudinal ‘social sensing’. Other studies along this line have shown that mobile phones can be used effectively to sense aspects of human spatial behavior [16] [17] [18] [19] and social interactions [20] [21] [22]. Thus, in this paper, we demonstrate how longitudinal mobile phone data can allow us to empirically examine the relationship between weather conditions and social behavioral patterns.

## Methodology

### Ethics Statement

This research was granted ethical approval from the IRB at Newcastle University, UK. The consent was not needed for this study because the data used in the study was anonymized by the operator before being transferred to the authors, in such a way that no connection could be made with any individual. The mobile phone users agreed to the terms and conditions for using the services with the network operator that their communications may be recorded and analyzed for improving services.

### Datasets

**Figure 1:**
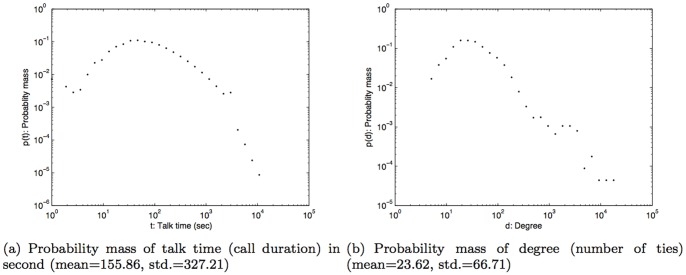
Probability mass of talk time and degree.

In this study, we used anonymized Call Detail Records (CDR) of mobile phone users in Lisbon, Portugal over the course of one year: from April 2006 to March 2007. The data was collected for billing purposes by an European telecom operator. To safeguard personal privacy, individual phone numbers were anonymized by the operator before leaving their storage facilities, and were identified with a security ID (hash code). The CDR comprised the voice call information: *timestamp*, *caller's ID*, *callee's ID*, *call duration*, *caller's connected cellular tower ID*, and *callee's connected cellular tower ID*. The dataset did not contain information relating to text messages (SMS) or data usage (Internet). The location of the mobile phone user was recorded as the nearest connected cellular tower location when the user made or received a call. The dataset provided us with social and spatial characteristics of the mobile phone users over an extensive temporal window of observation. From the original dataset of over 1.3 million mobile phone users in Portugal, only the subjects, whose home and work location were estimated to be in the Lisbon study area, were selected for this study. The home and work locations were estimated based on the spatial distribution of the call activity intensity during nighttime and daytime [16]. The home and work locations estimated using this method have been shown to be reasonable proxies of human geography based on a comparison study using the Portuguese census data [16]. This led to the consideration of 22,696 subjects from the dataset, which accounted for 53,210,299 CDR for this study. Following [23] we only considered reciprocal communications in inferring the social network for each subject. On average, each subject was connected to about 24 different social ties (degree) and spent about three minutes on a phone call. The probability mass function of the subjects' talk time (call duration) and degree are shown in Fig. 1.

The other set of data used in this study was a historic record of weather conditions, which was obtained from the Weather Underground Agency of Michigan (www.weatherunderground.com). The Weather Underground had been used as the weather provider for the Associated Press and weather reports for some newspapers (including the San Francisco Chronicle) as well as the Google search engine. Some researchers have also used Weather Underground's dataset in their investigations, e.g., [24] and [25].

**Figure 2:**
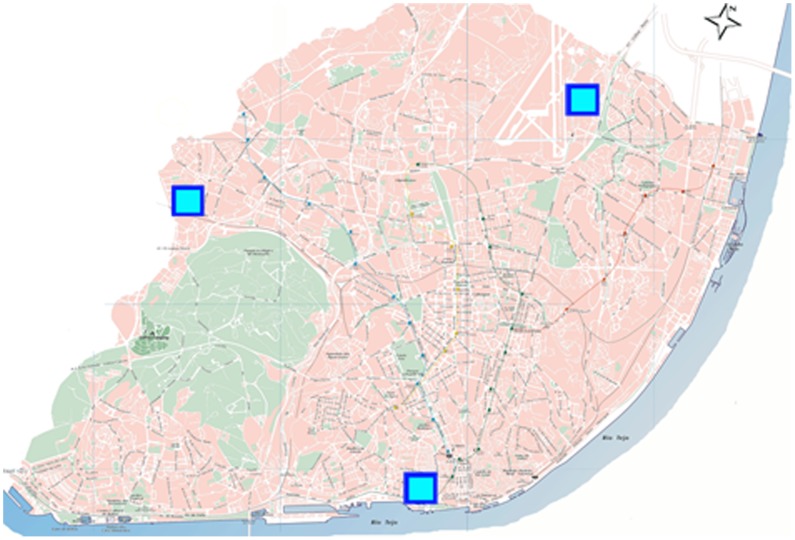
Locations of the weather stations in Lisbon, Portugal.

**Figure 3:**
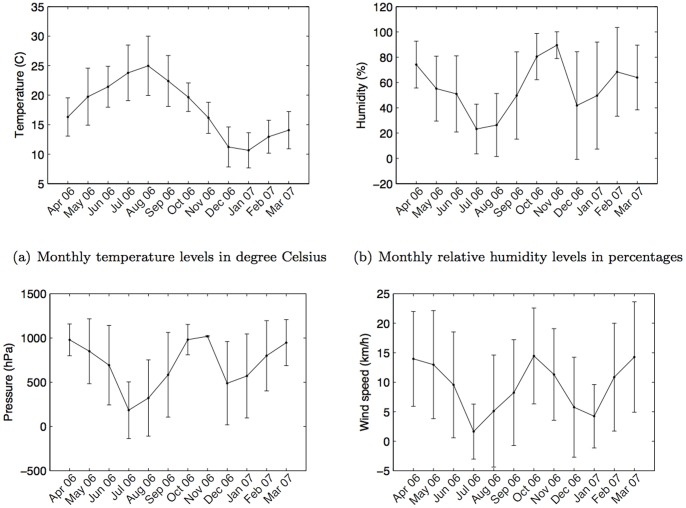
Monthly weather conditions in Lisbon during the period of analysis (April 2006–March 2007). *Note*: the standard air pressure is 1,013.25 hPa.

The dataset included logs of the climatic conditions: *temperature* (°C), *relative humidity* (%), *air pressure* (aPh), and *wind speed* (km/h), every 30 minutes from three weather stations situated in different locations in the city: Lisbon International Airport, Portela, and Cais do Sodre (as shown in Fig. 2). The average weather conditions (monthly) during the period of the analysis in this study (April 2006–March 2007) are shown in Fig. 3 with standard deviation bars. For our analysis, weather condition values were taken as the average of values recorded from all three weather stations. For example, the value of the temperature at a given time from the three stations is reported as an average temperature for that given time in this study. The average distance between cell tower and weather station is 747.5 meters.

### Analysis

Social interactions refer to the interactions between two or more individuals. In mobile social networks, the sociability of an individual can be estimated based on the overall amount of time spent (talking) on the mobile phone, and the number of people in his/her personal network. In addition, the strength of social relationship can also offer an insight into the type of social ties (e.g., strong ties, weak ties) being connected under different situations (e.g., weather conditions).

To quantify a social link's strength, we adopted the theory developed by Mark Granovetter in his milestone paper of 1973 [26] in which he categorized ties as one of two types: *strong* and *weak*. Strong ties are people who are socially close to us and whose social circles tightly overlap with our own. Typically they are people we trust and with whom we share several common interests. By contrast, weak ties represent mere acquaintances. Granovetter defined the strength of a tie as “a combination of the amount of time, the emotional intensity, the intimacy (mutual confiding), and the reciprocal services” ([26, p. 1361]). We adopted a similar approach to [23] using the amount of time spent in communication and reciprocity as proxies. By computing tie strength based on call duration normalized by the average as given by Eq. (1) we can categorize ties accordingly.
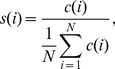
(1)where 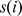 is the tie strength with tie 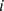, 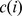 is the total call duration with tie 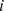, and the denominator is the average call duration of all associated ties where 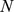 is the number of associated ties. We then classify ties for which 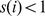 as *weak ties* while *strong ties* are ties for which 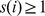.

In this study we investigated the relation between weather condition on social interactions by using *call duration* (time spent on phone calls), *degree* (number of connected social ties), and *kind of social tie* (the strength of connected ties) as indicators of social interactions, and using the climatic condition data collected from three different locations of the weather stations in Lisbon (*temperature*, *relative humidity*, *air pressure*, and *wind speed*) as our weather parameters.

## Results

Although certain weather parameters depend upon others, in this study we considered the effect of each weather parameter on individual indicators of social interactions. The following results show how the range of each weather parameter correlates to individual indicators of social interactions – i.e., we retrieved CDR and examined the distribution of each indicator of social interaction under specific weather conditions.

### Weather effect on call duration

Talking with people on a mobile phone is a way of maintaining and developing social relationships [20] and is therefore a good indicator of social relation strength [26]. To find out how the weather impacts conversation time, we examined the statistical distribution of call durations similar to Fig. 1 for different ranges of each weather parameter. To make the analysis easier and to allow us to more easily discern any emergent patterns we created a set of ranges, or bands, for each weather-related parameter. Based on the climate history (Fig. 3), temperature was considered between 5°C and 35°C and grouped into six bands, each with a 5-degree span; humidity was evenly divided into five bands in the range from 0% to 100%; air pressure in six even bands between 990–1050hPa; and wind speed in four bands between 0–20 km/h.

The statistical distribution of the talk time for each level of each weather parameter was computed as a *probability mass function* (pmf). This pmf is a normalized histogram on the logarithmic scale, where normalization allows comparisons across different bands of each weather parameter, and a logarithmic scale was used because of the nature of the statistical distribution of the call duration [27]. The pmf is a discrete probability distribution and its value represents the likelihood of a random variable (in our case call duration).

**Figure 4:**
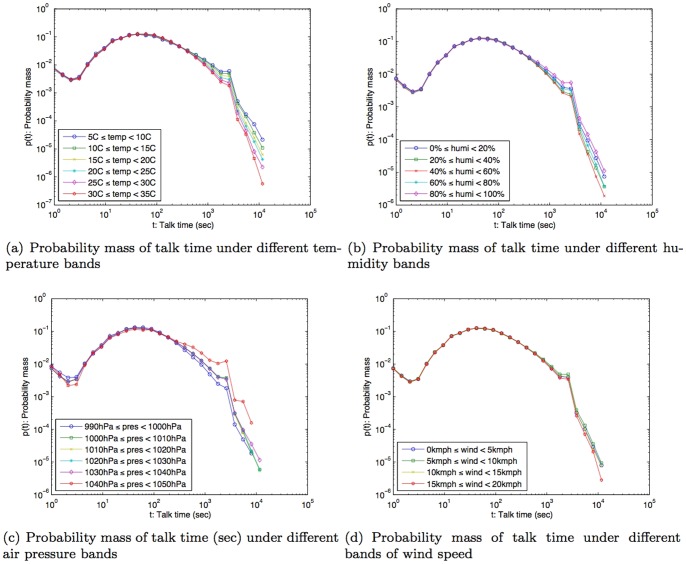
Probability mass of talk time under different weather conditions.

The results show that the average call duration does not vary greatly when compared to different environmental temperatures (Fig. 4(a)). However, the lower the temperature (i.e., as temperature drops), we notice an increase in the likelihood for phone conversations to be longer than six minutes. In other words, there is a correlation between the drop in temperature and the likelihood that a call will be longer than six minutes. This result suggests that the colder the day, the more likely that people would make longer phone calls. The humidity level exhibits a similar correlation on conversation duration. Again, while different humidity levels did not influence the average duration of phone conversation, there is an increase in likelihood for conversations to be longer than six minutes when the humidity is either very high (80%–100%) of very low (0%–20%) (Fig. 4(b)). Air pressure also does not appear to influence peoples' average call duration. However, at very high air pressures, i.e., above 1,040hPa (where 1,013.25 hPa is the normal air pressure), there is an increase in likelihood for phone calls to last longer than the average three minutes (Fig. 4(c)). Wind speed, on the other hand, does not appear to have any much influence upon talk time (see Fig. 4(d)).

**Figure 5:**
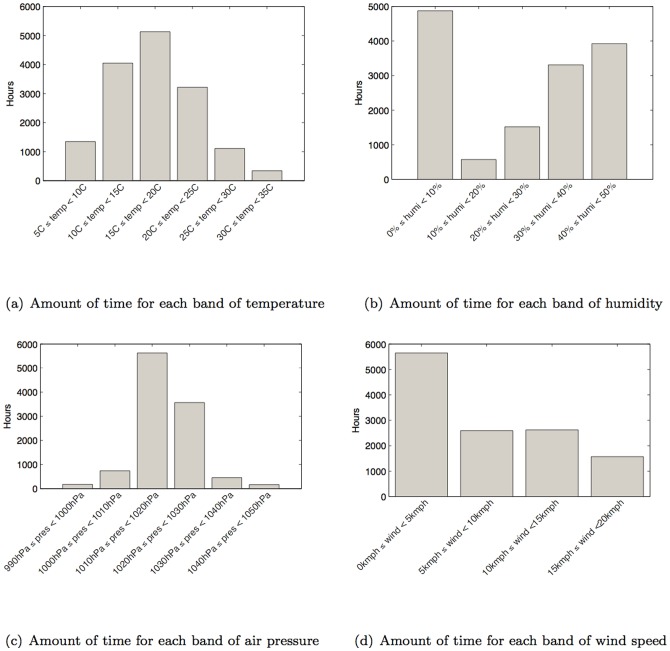
Time (in hours) for each band of weather parameters considered in this study.

In summary, the individual weather parameters do not appear to impact on the average duration of people's phone calls (which is around three minutes), but under certain weather conditions, we found an increase in the likelihood for people to make longer calls, i.e., calls longer than six minutes. Colder weather or extreme humidity appeared to increase the likelihood of longer call durations. Days of very high air pressure conditions also appeared to increase the likelihood of above average call durations while the wind speed appeared to show no influence. The time (in hours) for each band of the weather parameters experienced by the people in this study is shown in Fig. 5.

### Weather effect on degree (number of social ties)

Typically we connect to a number of different people using our mobile phone and for a range of purposes. The number of people in an individual's mobile phone network can be used to understand the structure of that individual's social network [28], and this can also facilitate many applications such as prediction of mobile phone viruses [29] and the assessment of socioeconomic opportunity [30]. Here we attempted to see the correlation between the change in weather conditions and the number of social ties people connect to. Using the same approach to understand the influence of weather conditions upon the duration of phone conversations, we examined the correlation between degree and different weather parameters. For example, the number of social ties people connect to for different bands of temperature. Because the observation time lengths of each weather parameterÕs bands were different (as shown in Fig. 5), we decided to normalize the number of ties in relation to time into ‘ties per hour’. This ensures that the results would not be influenced by the differences in observation time lengths.

**Figure 6:**
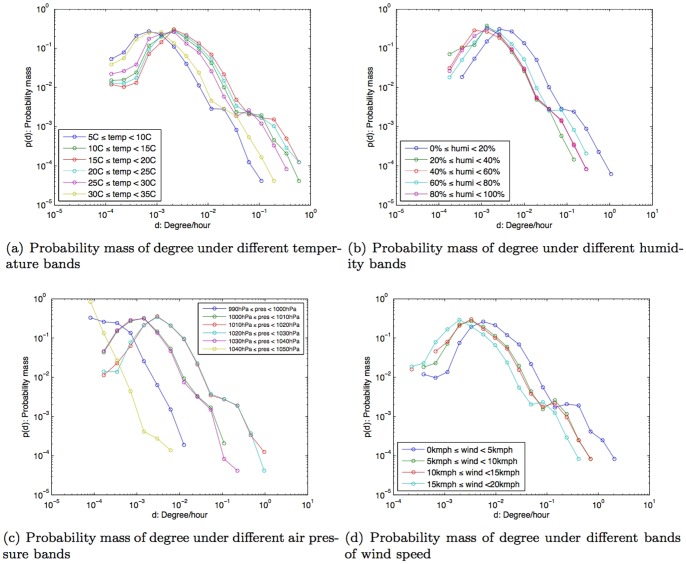
Probability mass of degree/hour under different weather conditions.

We observed that at the extreme bands of temperature (5°C–10°C) and (30°C–35°C), people tend to connect to fewer number of social ties when compared to other temperature bands (as shown in Fig. 6(a)). In other words, people tend to be less connected with their social ties during either very cold or very warm temperatures (the annual temperature range of Lisbon from our dataset range from 5°C–35°C). We observed a similar correlation with the humidity level. At humidity bands of 20%–100%, people also tend to be connected with fewer people within their mobile social network (as shown in Fig. 6(b)).

As for the air pressure, we found three distinct patterns. Firstly, for the air pressure band of 1010hPa–1030hPa (the band where normal air pressure falls into), people were found to be likely to be connected to 0.004 social ties/hour. Secondly, at air pressure bands of 1000hPa–1010hPa and 1030hPa–1040hPa (which are respectively lower and higher than the normal), we found that people were connected to less number of their social ties, around 0.002 ties/hour. Thirdly, at extreme air pressure bands (990hPa–1000hPa and 1040hPa–1050hPa), we found that people connected to the least number of their social ties, around 0.0004 ties/hour. This can be seen in Fig. 6(c)). In terms of wind speed conditions, the result in Fig. 6(d) shows that on days where there is little or no wind (0–5 km/h), people were found to be connected to 0.006 ties/hour. However, when wind speed was stronger than 5 km/h, connection to social ties were found to drop slightly, down to 0.002 ties/hour.

To summarize, under extreme weather conditions that might lead to feelings of discomfort, people were found to interact with fewer social ties via their mobile phones.

### Weather effect on the kinds of connected social ties

The results so far suggest that the weather condition has an influence on the time people spent talking on their mobile phone and the number of people they communicate with. We have learned that colder temperature, extreme humidity, and high air pressure can lead to an increase in the likelihood of long phone calls (i.e., more than six minutes), and at the same time extreme weather conditions were found to reduce the number of contacted social ties. With this in mind, we wanted to find out what kind of social ties people maintained connections to during those presumably uncomfortable weather conditions. We did this by applying the concept of tie strength (as developed by Granovetter [26] and Onnela et al., [23]) to work out the number of strong and weak ties in people's mobile phone network described previously (in the analysis section).

**Figure 7:**
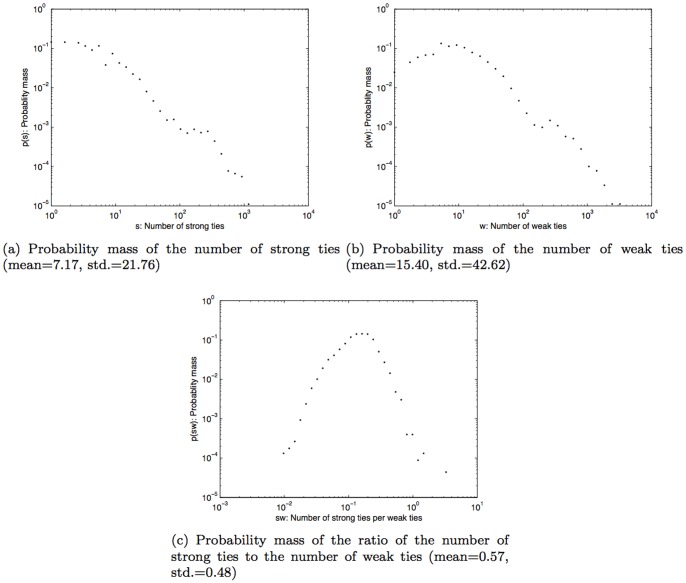
Probability mass of the number of strong ties, weak ties, and the ratio of the number of strong ties to the number of weak ties.

**Figure 8:**
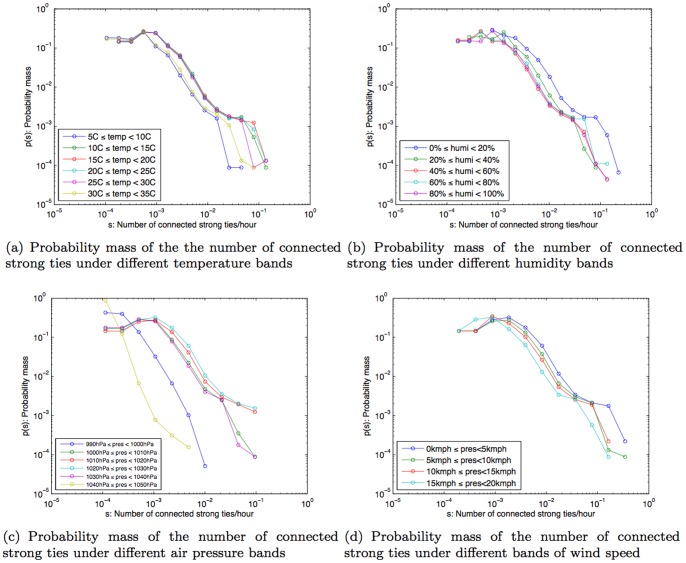
Probability mass of the number of connected strong ties/hour under different weather conditions.

The overall distributions of strong and weak ties in our dataset are shown in Fig. 7(a) and (b) respectively. It turns out that peoples' connectedness to the number of strong ties within their network is not greatly influenced by the temperature, humidity, or wind speed. However we found a more noticeable correlation with extreme air pressure conditions (990hPa–1000hPa and 1040hPa–1050hPa). In such conditions, people were connected with fewer strong ties as shown in Fig. 8.

**Figure 9:**
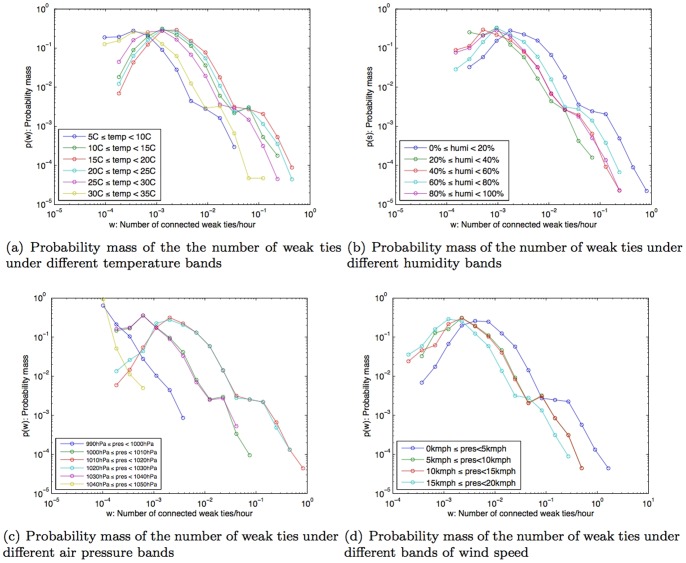
Probability mass of the number of weak ties/hour under different weather conditions.

On the other hand, the influence of weather is more noticeable for peoples' connectedness to weak ties within their network (Fig. 9). We found that the number of weak ties people were connected to, decreased under weather conditions that may be uncomfortable for people. This was observed with extreme temperature bands such as (5°C–10°C) and (30°C–35°C). Humidity bands of 20%–100% showed the same correlation. When there is little or no wind (0–5 km/h), we found that people were connected to 0.008 weak ties/hour via their mobile phones. When wind speed was stronger than 5 km/h, their average connectedness to weak social ties was found to drop to 0.001 ties/hour.

For air pressure, we discerned three distinct patterns. Firstly, for air pressure band of 1010hPa–1030hPa, the band that normal air pressure falls into, we did not see any differences in the number of weak ties that people were connected to. Secondly, at the air pressure bands of 1000hPa–1010hPa and 1030hPa–1040hPa (which are respectively lower and higher than the normal), we found peoples' connectedness to their weak ties decreased when compared to the condition when air pressure is normal. Thirdly, at extreme air pressure bands (990hPa–1000hPa and 1040hPa–1050hPa), we found that people were connected to very few weak ties (about 0.002 ties/hour).

Given that the number of connected ties were found to decrease with weather conditions that are uncomfortable, we then sought to determine whether weak ties or strong ties were more influenced by uncomfortable weather conditions. This is because, as we have shown earlier, the total number of strong ties that people were connected to was less than the number of connected weak ties (as shown in Fig. 7(a,b) and also evidenced in [22] [28]). Thus, to understand whether uncomfortable weather conditions had a stronger influence on connectedness to either kinds of ties, we had to compare the ratio of the number of connected strong ties to the number of connected weak ties under each weather parameters. The overall ratio of the relationship between strong ties and weak ties is shown in Fig. 7(c). Note that at this stage, this figure does not yet reflect the influence of different weather parameters.

**Figure 10:**
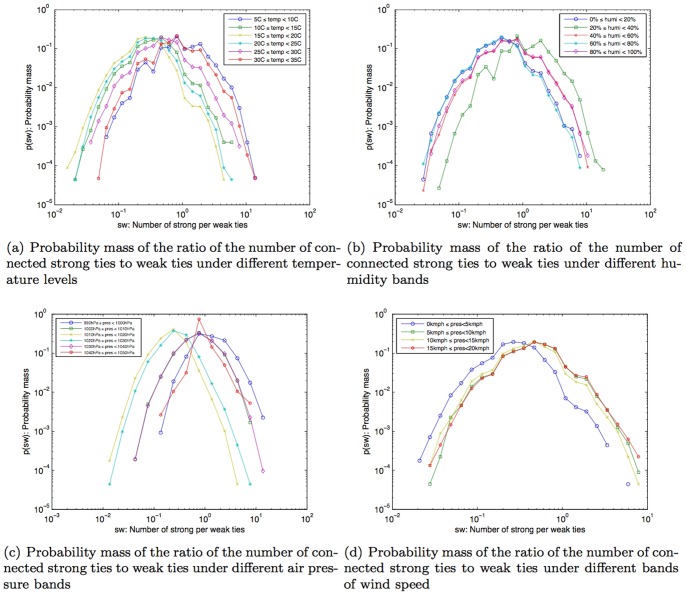
Probability mass of the ratio of the number of connected strong ties to weak ties under different weather conditions.

We then computed the ratio of this relationship with each different weather parameter. The results in Fig. 10 show us that although people were connected to fewer social ties when the weather conditions were uncomfortable, people lost a higher proportion of connectedness with weak ties when compared to their connectedness with strong ties.

In summary, under presumably uncomfortable weather conditions, i.e., very cold, very warm, very high air pressure, very low air pressure, or windy conditions, people are more likely to interact with fewer social ties within their mobile phone network but the connection loss of the weak ties was higher than that of the strong ties. This result is also in line with the findings of [22], which established that the social interaction with strong ties is less dependent upon extrinsic factors than weak ties.

## Conclusions

The ubiquity of personal communication technologies, such as mobile phones, means that these technologies can themselves be used to study the relationship between external factors, such as weather conditions and social behavior. We used a number of proxies for social interactions, including time spent on phone calls, number of connected social ties, and the strength of those connected ties. We considered the relationship between these proxies and a number of weather factors including temperature, relative humidity, air pressure, and wind speed. By analyzing our longitudinal data, which includes a full calendar year of communication records for 22,696 mobile phone users (53.2 million call logs), we have discovered a number of interesting results (result summaries are given in Tables 1); our main findings are as following:

- weather conditions were found to have a correlation with mobile phone social interactions;
- weather conditions do not greatly influence people's average call time;
- as the weather becomes increasingly cold, or when the air pressure is high, the likelihood of longer calls (more than six minutes) increases;
- under (presumably) uncomfortable weather conditions (i.e., very cold/warm, very low/high air pressure, and/or windy) people tend to communicate with fewer social ties;
- although people interact with a smaller number of social ties during uncomfortable weather conditions, they tend to maintain their connections with strong ties more than with weak ties.

**Table 1:** Summary of results.

	Talk Time	Degree	Tie Strength
Temperature	No effect on average talk time. Colder weather increases the likelihood of call duration to be longer than 6 min.	Under extreme temperature conditions (very cold, very warm), people tend to talk to a smaller number of social ties.	Under extreme temperature conditions (very cold, very warm), people tend to connect to a smaller number of social ties. However, they maintain their connections with strong social ties more than with weak ties.
Humidity	No effect on average talk time. Under extreme humidity (very high, very low) there is an increased likelihood for people's call time to be longer than 6 min.	Under humidity bands of 20%–100%, people tend to talk to a smaller number of social ties.	Under humidity bands of 20%–100%, people tend to connect to a smaller number of social ties. However, they maintain their connections with strong social ties more than with weak ties.
Air Pressure	No effect on average talk time. Very high air pressure increases the likelihood of calls to be longer than 6 min.	Under extreme air pressure conditions (very high, very low), people tend to talk to a smaller number of social ties.	Under extreme air pressure conditions (very high, very low), people tend to connect to a smaller number of social ties. However, they maintain their connections with strong social ties more than with weak ties.
Wind Speed	No effect on talk time.	Under windy condition (when wind speed becomes stronger than 5 km/h), people tend to talk to a smaller number of social ties.	Under windy condition (when wind speed becomes stronger than 5 km/h), people tend to connect to a smaller number of social ties. However, they maintain their connections with strong social ties more than with weak ties.

Nonetheless, there are some limitations to the observations we present in this study. With differences in weather conditions in different parts of the city, the weather information gathered from the three weather stations used in the study may not in reality represent the actual weather conditions experienced by the people in this study. In addition, these people might be indoors and thus, shielded from certain types of weather conditions such as wind and extreme temperatures. Although different weather parameters work in complex ways to influence actual weather, we did not consider this overall complexity in the current study. Future studies can take this into consideration in order to obtain a more detailed understanding of the overall effects of weather. The effect of special events such as holidays, national and social events were not considered in the study. These events may have some influence on the results through the number and duration of calls made. This too remains to be explored in our future studies. The weather conditions of the observed individual and the person they are connected to will also be taken into account. Finally, the mobile data offered insights into only a small part of the overall individual's sociability. The results obtained in this study are within the constraint of our tie strength calculation. Our future research direction will need to consider the interplay of weather parameters and other extrinsic factors that may also have an impact on social interaction such as the day of the week or weekly activity patterns, time of the day, holidays, vacations, and special social events.

This study does offer another way to understand how and under what conditions weather have an influence upon our use of the mobile phone to connect with our social network. With further elucidation, the findings we present can offer interesting insights to a number of domains such as the detection of the spread of mobile phone viruses [29], modeling information dissemination in social networks [31], designing recommendation systems [32], and of course, marketing [33]. More importantly, it can add to other studies that can further their investigations and understanding into the effects of weather on our moods, physical self, and even socioeconomic behavior [6] [30].

In itself, the case we present can open up new trajectories for research. For one, such high level and interesting correlations (as well as insights) offer researchers justifications for follow up research that are more focused and that dwells more deeply into these observed ‘relationships’. For example, a deeper investigation of why people are more likely to talk longer on mobile phones on colder days but predominantly with those of strong ties, could reveal not only the motivation, the actual relationships but also people's experiences of the connectivity during these cold days. More interestingly, are there any, and if so, how does this correlate with people's interactions with other communication technologies and social media? Establishing a more detailed understanding of such questions may inform an understanding of the dynamic rhythms of social bonds, people's (subconscious) needs and experience of connectedness under particular weather parameters. This could also elucidate the range and roles of the communication technologies involved as well as to what extent they could support people's sense of well-being.

Secondly, this work could enrich and inspire our design of future technologies, especially social technologies. For instance, while context-aware computing is good at sensing our physical and increasingly our social environment, we don't believe that designers have thought about the need to sense different weather parameters. By adding the awareness of weather parameters and how they shape people's emotional needs and experience for-and-of social connectivity to future technologies, such technologies may not only be easy and efficient to use but could also be more supportive of how we feel and of our sense of well-being.

Finally, the approach we present in this paper can be a useful tool for Human-Computer Interaction (HCI) researchers (and social scientists) who are interested in explicating the role of mobile phone use, the need for connectivity and its influence upon people's sociality in general. This large-scale data-centric approach can complement their research that tends to work more deeply with the phenomenon and with fewer numbers of people. By looking into the sensor-based data of large populations that are increasingly easily and readily captured, HCI researchers could get broad indications of (emergent) relationships, unexpected juxtapositions, and a sense of the floor to ceiling effects or overall trends of particular social phenomenon. In addition, by analyzing various combinations of such large datasets, they might also identify novel or interesting social phenomena that are worth pursuing in greater depth.
